# Chronic sublethal stress causes bee colony failure

**DOI:** 10.1111/ele.12188

**Published:** 2013-10-06

**Authors:** John Bryden, Richard J Gill, Robert A A Mitton, Nigel E Raine, Vincent A A Jansen, David Hodgson

**Affiliations:** 1 School of Biological Sciences, Royal Holloway University of LondonEgham, Surrey, TW20 0EX, UK; 2 Department of Life Sciences, Imperial College London, Silwood Park campusAscot, Berkshire, SL5 7PY, UK

**Keywords:** Environmental stressor, imidacloprid, mathematical model, neonicotinoid pesticide, pollinator decline

## Abstract

Current bee population declines and colony failures are well documented yet poorly understood and no single factor has been identified as a leading cause. The evidence is equivocal and puzzling: for instance, many pathogens and parasites can be found in both failing and surviving colonies and field pesticide exposure is typically sublethal. Here, we investigate how these results can be due to sublethal stress impairing colony function. We mathematically modelled stress on individual bees which impairs colony function and found how positive density dependence can cause multiple dynamic outcomes: some colonies fail while others thrive. We then exposed bumblebee colonies to sublethal levels of a neonicotinoid pesticide. The dynamics of colony failure, which we observed, were most accurately described by our model. We argue that our model can explain the enigmatic aspects of bee colony failures, highlighting an important role for sublethal stress in colony declines.

## Introduction

The social bees (honeybees, bumblebees and stingless bees) provide an important ecosystem service, pollinating both wildflowers and agricultural crops (Klein *et al*. 2007; Winfree *et al*. 2008; Potts *et al*. 2010). Reports of social bee colony losses (Oldroyd 2007; Ratnieks & Carreck 2010) and global bee population declines (Biesmeijer *et al*. 2006; Goulson *et al*. 2008; Brown & Paxton 2009; Cameron *et al*. 2011) are therefore of major concern. Several stressors have been implicated in bee declines (Vanbergen & The Insect Pollinators Initiative 2013), including pesticides (Desneux *et al*. 2007), disease and parasites (Brown & Paxton 2009) and habitat change and loss (Kremen *et al*. 2007; Potts *et al*. 2010). While there is still debate over which stressors are most detrimental, no single factor has emerged as an overall primary cause (Ratnieks & Carreck 2010; Vanbergen & The Insect Pollinators Initiative 2013).

Recent evidence has indicated that many environmental stressors can affect bees even when they do not cause direct mortality (so called *sublethal* impact). For instance, exposure to field level pesticides can affect worker mobility, memory, orientation and foraging performance (Desneux *et al*. 2007; Gill *et al*. 2012; Schneider *et al*. 2012; Williamson & Wright 2013), and parasites can impose energetic stress (Moret & Schmid-Hempel 2000; Mayack & Naug 2009) as well as impair both learning (Iqbal & Mueller 2007) and thermoregulation (Schafer *et al*. 2011). While these sublethal effects need not kill individual bees, they may have profound effects on the dynamics and functioning of the whole colony. Despite this, we currently have little understanding of how chronic sublethal stress, experienced by individual bees, can cause colonies to fail.

There is an important difference between the effects of lethal and sublethal stress when considering the functioning of social bee colonies. Redundancy in bee colonies allows them to lose a significant proportion of their worker force without any apparent significant impact on colony function and productivity (Schmid-Hempel & Heeb 1991; Müller & Schmid-Hempel 1992; Rueppell *et al*. 2009). However, if bees become impaired rather than die, the impairment may impose a load on the colony and lead to a cumulative effect on normal colony function. Indeed, there is evidence that pesticide induced behavioural impairment can detrimentally feedback to colony eclosion (birth) and death rates (Gill *et al*. 2012), the production of sexuals (Whitehorn *et al*. 2012) and increase the prevalence of disease (Brown *et al*. 2000). This feedback onto birth and death rates generates Allee effects which lead to colony dynamics that are uncertain, such as those with multiple outcomes and breakpoints (May 1977). We show here that effects of sublethal stress on colony function are important to explain colony dynamics. We did this by formulating a mathematical model for colony dynamics that includes colony function. We subsequently fitted this model to novel empirical data from bumblebee colonies that were sublethally exposed to a pesticide and show that the description of this model is superior to models which do not account for impairment to colony function.

## Materials and methods

### Modelling

Our model accounts for SubLethal Stress (henceforth, the SLS Model) and describes healthy bees (*S*) and impaired bees (*I*) to represent behavioural impairment by a sublethal stressor. Healthy bees become impaired at rate *β*, and the level of behavioural impairment is expressed by the parameter *c *≤ 1 which reflects the reduced contribution of the impaired bees to colony function, so that the effective operational size of the colony is *N = S + cI*. The model describes a growing colony, where the eclosion rate of new bees in the colony is *bN*, with *b* as a rate constant. To capture individual effects of stress, known to increase the mortality rate of bees (Cartar & Dill 1991; Brown *et al*. 2000; Dainat *et al*. 2012), we introduced mortality of impaired bees at a constant rate *ν* to our model. We assume that the per capita death rate is inversely proportional to the effective size of the colony, *μ*/(*N *+ *φ*), where decreasing *φ* sets how sharply the death rate increases at low effective colony sizes and *μ* adjusts the overall rate. This reflects that colonies containing more impaired bees are less good at maintaining essential colony functions (e.g. foraging, thermoregulation, defence and hygienic behaviour). This gives the following equations:
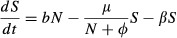
1
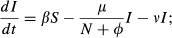
2

To visualise the dynamics of the SLS Model, phase plots were generated by numerically integrating the model over time and recording the trajectories of the variables. Many numerical runs were done, iterating over different initial values of *S* and *I* while keeping all other parameters the same. Each run was over a fixed time period (20 days).

The feedback of stressed bees onto colony function in the SLS Model distinguishes it from other mathematical models that model disease and increased mortality in bee colonies (Schmid-Hempel 1998; Sumpter & Martin 2004; Khoury *et al*. 2011, 2013; Becher *et al*. 2013). To assess the validity of the SLS Model, we compared it with three other alternative models (their equations are listed in Table 1) summarised as follows:

1. A model described by Khoury *et al*. (2011), that we therefore dub the Khoury model, describes how colony dwelling bees can transition between hive and forager roles. The model also describes the rate at which hive bees eclose and the rate at which forager bees experience mortality, see Khoury *et al*. (2011) for a full description. It has been used previously to describe colonies treated with pesticides (Henry *et al*. 2012).
2. The SLS Variant Model is similar to the SLS Model, but has a linear per capita death rate which is fixed at *μ* rather than *μ*/(*N *+ *φ*). It will be used as a comparison to test the importance of the nonlinear death rate term which introduces positive density dependence in the SLS Model.
3. The final model is the Larvae Adult Model (henceforth the LA Model) which considers that the pesticide could have a toxic effect on larvae, increasing its death rate. It models larva (number *L*) and adult bees (of number *A*). Larvae hatch at rate *g,* die at rate λ, eclose into adult bees at rate γ and adult bees die at rate ξ.

**Table 1:** The four models we fitted against the empirical data from the experimental bumblebee colonies

Model	Equations
SLS Model	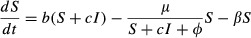
	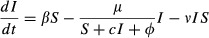
Khoury Model	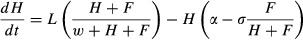
	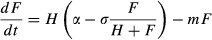
LA Model	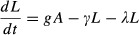
	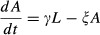
SLS Variant Model	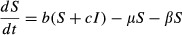
	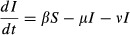

### Colony experiments

We provided bumblebee (*Bombus terrestris*) colonies a specified amount of sucrose solution (every 2 days and 3 days over the weekend) containing a sublethal concentration of a systemic neonicotinoid pesticide (10 ppb imidacloprid, see Supporting Information for further details). Neonicotinoids are used extensively on horticultural plants and flowering agricultural crops throughout the world. Imidacloprid is applied to crops such as oilseed rape (canola), sunflower, maize, linseed, peas and beans, cucurbits and orchards which are key food sources for bees (Cresswell 1999; Thompson 2001; Holzschuh *et al*. 2011). The concentration of imidacloprid that we used in this experiment was 10 ppb, which falls near the upper end of the field realistic range reported for nectar and pollen in agricultural crop species (Bonmatin *et al*. 2003, 2005; Chauzat *et al*. 2006, 2009; Krischik *et al*. 2007; Cresswell 2011; Blacquiere *et al*. 2012). We also provided colonies with a specified amount of untreated pollen every 2 days (3 days over the weekend). All colonies were provided with increasing levels of pollen and sucrose throughout the experiment to account for increasing demand as the colonies grew (see Supporting Information Section 1.2 for more details on pesticide treatment and feeding regime).

Colonies were housed in wooden nest boxes isolated from one another, and the outside world, in laboratory conditions throughout the experiment. We recorded the growth and development of 16 early-stage queen-right colonies (eight control colonies and eight pesticide-exposed colonies). To eliminate any bias, colonies were sorted by size at the start so that there was no significant difference in the number of workers (mean ± SEM: control = 6.5 ± 0.8; neonicotinoid = 8.25 ± 1.64; *t*-test: *t* = −0.96, *P *= 0.36) or pupae (mean ± SEM: control = 6.4 ± 1.8; neonicotinoid = 6.8 ± 1.53; *t*-test: *t* = 0.16, *P *= 0.88) per colony between control and neonicotinoid assigned colonies (see Supporting Information Section 1.1 for more details on the colony set-up). *B. terrestris* has relatively small colonies, compared to honeybees, making it possible to track all individual eclosions and deaths. Throughout the 42-day experiment, all colonies were inspected on a day-to-day basis to record the numbers of new eclosions and dead bees (see Supporting Information Section 1.3 for more details on experimental measurements).

### Model fitting

Our data collected from the experimental colonies consisted of day-to-day observations of the numbers of bees that had newly eclosed and died in each colony. We assessed four models against these data (see Table 1). To fit a colony’s data to solutions of the models, we used discrete stochastic versions of the models with a time interval of one day. The numbers of bees that eclose, die or transition between the two states of each model (the SLS Model and SLS Variant Model each have two variables being Susceptible and Impaired; the Khoury Model has two variables being Hive and Forager; the LA Model has two variables being Larva and Adult) were drawn from a Poisson distribution with means according to the corresponding rates in the deterministic models.

We used the Numerically Integrated State Space (NISS) algorithm to calculate a likelihood for a model and a solution given the data for eclosion and death rates for each colony algorithm (De Valpine & Hastings 2002). The NISS algorithm calculates the probability of observations for each time step *P*(*O*_*t*_), given the observations at the previous time step *P*(*O*_*t-1*_), a model and a solution. This is done by using the model to generate probabilities of all possible states at time step *t*, given all possible states at the previous time step *t −* 1. Given states of the model, probabilities of observations were calculated by summing the Poisson mass functions for the death and eclosion rates from the model for each state.

The log likelihood for a set of parameters was calculated by adding the logarithms of the probabilities of observations at every time step and for each colony. The maximum likelihood was then estimated using the adaptive Differential Evolution optimisation algorithm (Brest *et al*. 2006) with 80 solutions per generation. Optimisation stopped when 25 generations passed with no new best solution. The initial state of the model is given by the initial state of each experimental colony (number of workers) as follows: SLS Model, all initial bees were healthy so *S = number of workers, I = 0*; Khoury Model, colony split in half with equal numbers of hive and forager bees, *H *= 0.5 × *number of workers, F *= 0.5 *× number of workers*; LA Model, *A = number of workers*, the initial number of larvae per adult was set by a parameter optimised by the Differential Evolution algorithm giving *L *= *A *× *initial_larvae_per_adult*.

## Results

**Figure 1:**
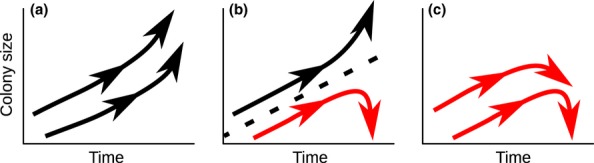
A schematic diagram showing the changing types of dynamics in the SLS Model due to an increasing rate of healthy bees becoming impaired (*β* increases over the three panels from left to right). Colonies with black trajectories grow whereas red trajectories lead to failure. (a) All colonies grow. (b) There is a breaking point (dashed line) between two basins of attraction depending on the numbers of bees present in the colony and the level of impaired bees in the colony; depending on initial conditions colonies will either grow or fail. (c) All colonies can grow at first but will eventually fail.

The SLS Model (Eqns 1 and 2) has fundamentally different dynamics for different values of the parameters. After an initial phase of growth the dynamics will show either: (1) growth for all colonies; (2) multiple outcomes with some colonies growing and others failing dependent on the initial conditions; or (3) all colonies decline and fail (see Fig.1; Supporting Information Section 2.1). The presence of multiple outcomes relies on the *μ*/(*N *+ *φ*) term (Supporting Information Section 2.2) which introduces positive density dependence in the model. With a simpler term (see SLS Variant Model) there is still a sharp boundary between growth (Fig.1a) and failure (Fig.1c).

**Figure 2:**
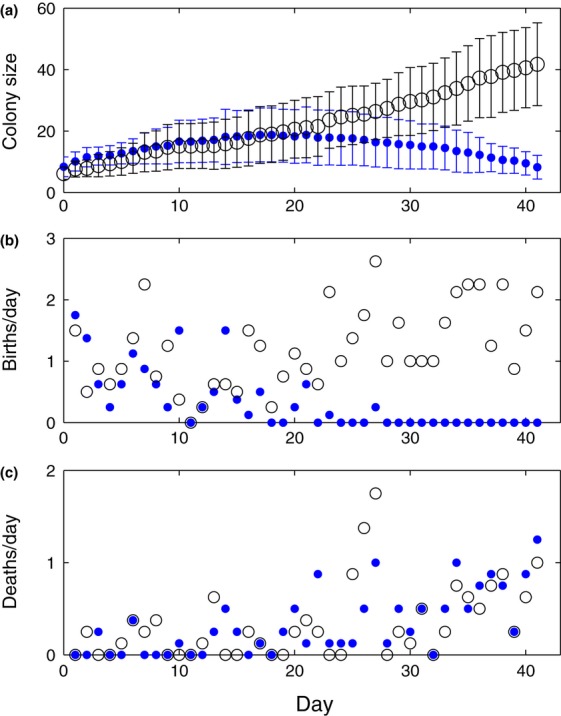
Comparison of neonicotinoid treated colonies (blue filled circles) with control colonies (black open circles). (a) Both treatment and control (plot shows mean numbers of workers ± 95% CI) have similar growth at first, but trajectories begin to diverge after approximately 3 weeks. (b, c) Worker birth and death rates (mean number of births and deaths per colony are shown) for treatment and control colonies illustrate the impact of the pesticide on colony health. In the treatment colonies, the birth rate decreases while colony size increases during the first 21 days, and after 21 days the death rate increases while colony size decreases.

The pesticide treated colonies in our empirical experiment followed a similar growth pattern to that observed for SLS Model runs ending in colony failure. By the end of the 42-day experiment, we found a significant difference in colony size between control and treatment colonies (Fig.2 and data in Tables S1–S6). While all colonies grew at a similar rate during the first 3 weeks, only control colonies continued growing throughout the 42-day study, whereas treatment colonies began to shrink (at 33 days the average colony size of the neonicotinoid treated colonies was and continued to be significantly lower than control colonies; *t*-test for all comparisons: *P *< 0.05). Fig.2 also indicates that there is impaired colony function in the pesticide treated colonies because the birth rates decreased (relative to control colonies) while colonies were still growing, and the death rates also increased during the period in which treated colonies were in decline. This analysis of the data from our experiment thus directly shows that sublethal pesticide exposure decreases colony size after a lagged growth period, and also indicates that this may be due to effects of impairment on colony function rather than direct mortality.

**Figure 3:**
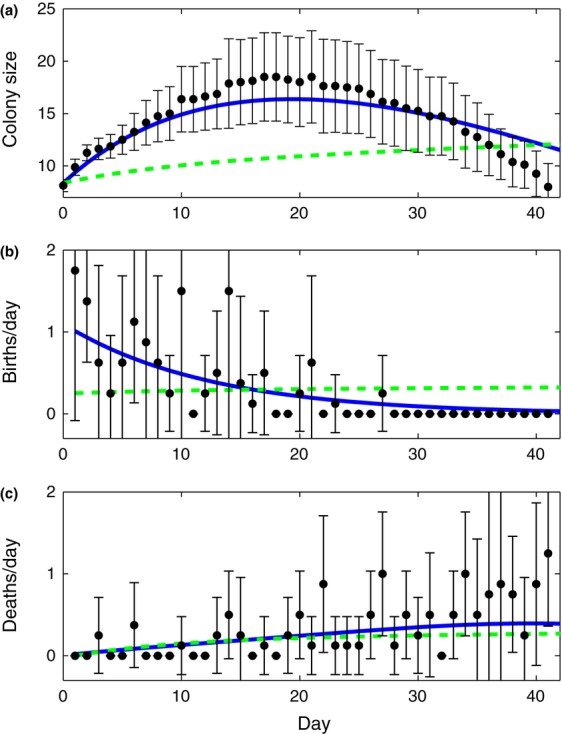
Pesticide treated colonies grew before going into decline. (a) The SLS Model (blue line), fits this dynamic well, while the Khoury Model (green dashed line) simply tends to equilibrium. (b, c) The fitting was done against birth and death rates. Data points show the mean (± SEM) across the eight colonies. Model curves show the mean of eight model runs, each starting as a healthy colony at the size of a corresponding empirical colony. Extensive tests varying starting conditions showed that all SLS Model runs have the same outcome of colony failure. SLS parameters: *b *= 0.126, *c *= 0.00332, *β *= 0.202, *ν *= 0.00625, *μ *= 0.0209, *ϕ* = 0.402. Khoury parameters: *L *= 0.690, *α *= 0.103, *δ *= 1.96 × 10^−7^, *m *= 0.0297, *w *= 13.9.

To assess whether colony function is important for explaining colony dynamics, we used a model fitting approach to investigate the patterns of colony sizes, birth rates and death rates found in the treatment colonies. We did this by comparing the fit of the SLS Model with two alternative models. The Khoury Model incorporates lethal stress, but not the impairment and feedback caused by sublethal stress (Khoury *et al*. 2011). The LA Model incorporates toxic effects from pesticides upon larvae, which could introduce delayed effects on colony size by killing larvae. We fitted the three models to the empirical data using the NISS algorithm (De Valpine & Hastings 2002), which calculates a likelihood value for a model based on all possible trajectories. Parameters were optimised using Differential Evolution (Brest *et al*. 2006). The best fits found for the SLS Model and the Khoury Model are shown in Fig.3 (see Fig. S2 for a comparison of the LA Model and SLS Model). Using Akaike weights we selected the best model, and found that the SLS Model describes the data overwhelmingly better with essentially no support for the Khoury Model or the LA Model compared with the SLS Model (Table 2). Of the three models tested, only the SLS model (that incorporates feedback of colony function on birth and death rates) matched the pattern of birth rates decreasing and death rates increasing in the treatment colonies.

**Table 2:** Summary of the models tested against the treated bumblebee colonies

Model	Log likelihood	AIC	Akaike Weight
SLS	−418	852	1
Khoury	−510	1034	0
LA Model	−446	904	0
SLS variant	−437	888	0

The table shows, for each model, the maximum log likelihood found together with calculated Akaike Information Criteria (AIC) values and Akaike weights. There is essentially no support (Burnham & Anderson 2002) for the Khoury Model, SLS Variant or LA Model compared with the SLS Model.

This leads us to conclude that colony function is a key element to explain the dynamics of our treatment colonies, and suggests an explanation for the mechanism by which sublethal effects lead to colony failure. Social bee colonies depend on the efficient cooperative performance of multiple individual workers so that essential tasks such as foraging, thermoregulation and brood care, sustain and enhance overall colony function. They have many workers and are able to buffer some effects of stress. However, if too many bees become behaviourally impaired, irrespective of the reason, the colony reaches a tipping point and is set on a path to failure through moderate, but chronic, levels of stress. If the stress level is below this tipping point, the colony can continue growing. There is, consequently, a critical stress level, where a small change in the amount of stress can mean the difference between colony growth or failure.

Next, we investigated whether there is evidence for positive density dependence in the dynamics of our colonies. To do so, we also fitted the data to a variant of the SLS Model in which we replaced the positive density dependence term *μ*/(*N *+ *φ*) with a linear death rate *μ*. The variant model does not allow for multiple outcomes (see Supporting Information Section 2.2), but there is still a critical stress level for colony failure when impairment is too high (Fig.1a and c). Because the best fit of the variant model was much worse than the full SLS Model (Fig. S3), with Akaike weights showing essentially no support for the variant model (see Table 2), we can decisively infer that social bee colony dynamics are subject to positive density dependence and thus capable of showing multiple outcomes.

**Figure 4:**
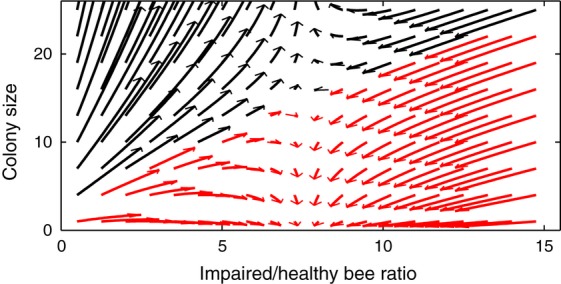
Phase plane demonstrating multiple outcomes in the dynamics of the SLS Model between colony growth and failure. Trajectories denote 20 days of the model starting from different initial conditions (see Methods for more information) and are depicted by arrows coloured according to whether they lead to continued growth (black arrows) or colony failure (red arrows). Parameters were the same as those in Fig.3 with *β *= 0.12.

The SLS Model predicts that the level of pesticide is a crucial factor in setting the dynamics of colonies. In our empirical experiment bees received an imidacloprid concentration, in their isolated colonies, near the upper range of that typically found in field realistic conditions (Cresswell 2011; Blacquiere *et al*. 2012). In a previous experiment (Gill *et al*. 2012), bumblebees were treated with the same concentration of imidacloprid, but we would expect them to have received a lower level of exposure as they were free to forage for both pollen and nectar in the field. The imidacloprid treated colonies continued to grow throughout the Gill *et al*. (2012) study, a dynamic consistent with lower exposure in the SLS Model (Fig.1a). In this study, treated colonies showed a dynamic consistent with higher pesticide exposure in the SLS Model (Fig.1c), as all treatment colonies failed. Our model predicts that multiple outcomes are possible at a mid-range of imidacloprid exposure. To simulate lower exposure in our model we can reduce parameter *β* from that found in the fit against the treatment colonies, keeping all other parameters the same, and we observe multiple outcomes in the model’s dynamics (see Fig.4).

## Discussion

We have shown here that bumblebee colonies fail when exposed to sustained sublethal levels of pesticide, and that this can be explained by a decrease in colony function. By testing a suite of models against data collected from failing colonies, we are able to make several inferences. We infer that social bee colonies have positive density dependence, they are subject to an Allee effect, and also that there is a critical stress level for the success of a colony such that a small increase in the level of stress can make the difference between failure or success.

Our results provide two explanations as to why it has been so difficult to explain colony losses. First, although we imposed a specific (pesticide) stress in our experiment, the argument about reduced colony function applies to any stressor that reduces the contribution made by individual bees to colony function. This suggests that multifactorial stress can cause colony failure (Vanbergen & The Insect Pollinators Initiative 2013), but that failure is the result of a critical stress level from the accumulation of multiple sublethal factors (e.g. disease, weather and anthropogenic influences) without the need for synergy between these effects. This can explain why finding the link between colony failures and a single specific stress factor has so far proved elusive.

Second, it appears that a number of potential causes for the failure of bee colonies have been dismissed because the presence of the stressor is not a good predictor of colony failure (Cox-Foster *et al*. 2007). On the basis of our model, we predict that two colonies, with similar stress levels can have divergent fates through the Allee effect caused by the feedback through colony function. If the effect is strong, one colony may be growing, while the other is set on a path to failure. Even if the effect is weak, random events (such as other stresses or demographic noise) can put similar colonies on divergent trajectories. This will weaken the correlation between the level of a stressor and colony failure. For example, the parasite *Nosema ceranae* can cause colonies to fail (Higes *et al*. 2008). However, while *N. ceranae* was detected in 100% of failing colonies, it was also present in 47% of non-failing colonies (Cox-Foster *et al*. 2007). Our results therefore suggest that correlation statistics should be used with caution for inferring causality.

Our study found that the best fitting model to our empirical data was capable of showing multiple outcomes where the fate of the colony can depend on its initial size; however, our experimental results did not directly display these multiple outcomes. It is known to be highly challenging to empirically demonstrate such multiple outcomes (Ives *et al*. 2008; Mumby *et al*. 2013). In our case, the difficulty could arise from there being a very precise level of stress at which multiple outcomes exist and since bee colony populations are subject to random occurrences, the level of colony replication required would make such experiments difficult to perform. Even in the event that such experiments could be successfully conducted, the results would still require model fitting (such as that done in this paper) to verify these colonies did not fail due to random chance (process error). The results of such an experiment would in essence simply reconfirm what we have already shown here. The evidence for multiple outcomes that we find here comes from the fact that the SLS Model incorporates feedback from the population density onto the death rate (the Allee effect). By showing that this nonlinear response is important, we expect that any other models that have as strong a fit to our data as the SLS Model, will also show critical stress levels and multiple outcomes.

This study demonstrates two key aspects of how stress on individual bees can disrupt colony function and lead to colony failure. First, a stressor must have a chronic impact (over a period of several weeks) before we see any noticeable effect: meaning that risk assessments of a stressor’s impacts must be over a similar time scale. Second, we show how a stressor that impairs colony function can cause an Allee effect which makes colonies especially susceptible to failure from stress at earlier points in their life cycles. This has important implications for the number of colonies that need to be tested to assess the impacts of stressors to adequately measure the proportion of colonies that fail.

The dominance of social bees as crucial pollinators stems primarily from their social organisation: large colony sizes are supported by the efficient coordination of tasks across group members, such that colony performance is better than a collection of uncoordinated individuals. It is intriguing that the social organisation that leads to the success of social bees may also be a key factor in their declines and colony failures.

## Author statement

JB, RJG, NER and VAAJ conceived the work; JB and VAAJ developed and analysed the models; JB fitted the models to the data; RAAM, RJG and NER designed and carried out the experiment; JB, RJG, NER and VAAJ wrote the paper.

## References

[b1] Becher MA, Osborne JL, Thorbeck P, Kennedy PJ, Grimm V (2013). Towards a systems approach for understanding honeybee decline: a stocktaking and synthesis of existing models. J. Appl. Ecol.

[b2] Biesmeijer JC, Roberts SPM, Reemer M, Ohlemuller R, Edwards M, Peeters T (2006). Parallel declines in pollinators and insect-pollinated plants in Britain and the Netherlands. Science.

[b3] Blacquiere T, Smagghe G, van Gestel CAM, Mommaerts V (2012). Neonicotinoids in bees: a review on concentrations, side-effects and risk assessment. Ecotoxicology.

[b4] Bonmatin JM, Moineau I, Charvet R, Fleche C, Colin ME, Bengsch ER (2003). A LC/APCI-MS/MS method for analysis of imidacloprid in soils, in plants, and in pollens. Anal. Chem.

[b5] Bonmatin JM, Marchand PA, Charvet R, Moineau I, Bengsch ER, Colin ME (2005). Quantification of imidacloprid uptake in maize crops. J. Agric. Food Chem.

[b6] Brest J, Greiner S, Boskovic B, Mernik M, Zumer V (2006). Self-adapting control parameters in differential evolution: a comparative study on numerical benchmark problems. IEEE Trans. Evol. Comput.

[b7] Brown MJF, Paxton RJ (2009). The conservation of bees: a global perspective. Apidologie.

[b8] Brown MJF, Loosli R, Schmid-Hempel P (2000). Condition-dependent expression of virulence in a trypanosome infecting bumblebees. Oikos.

[b9] Burnham KP, Anderson DR (2002). Model Selection and Multimodel Inference: A Practical Information-Theoretic Approach.

[b10] Cameron SA, Lozier JD, Strange JP, Koch JB, Cordes N, Solter LF (2011). Patterns of widespread decline in North American bumble bees. Proc. Natl. Acad. Sci. USA.

[b11] Cartar RV, Dill LM (1991). Costs of energy shortfall for bumble bee colonies: predation, social parasitism, and brood development. Can. Entomol.

[b12] Chauzat MP, Faucon JP, Martel AC, Lachaize J, Cougoule N, Aubert M (2006). A survey of pesticide residues in pollen loads collected by honey bees in France. J. Econ. Entomol.

[b13] Chauzat MP, Carpentier P, Martel AC, Bougeard S, Cougoule N, Porta P (2009). Influence of pesticide residues on honey bee (Hymenoptera: Apidae) colony health in France. Environ. Entomol.

[b14] Cox-Foster DL, Conlan S, Holmes EC, Palacios G, Evans JD, Moran NA (2007). A metagenomic survey of microbes in honey bee colony collapse disorder. Science.

[b15] Cresswell JE (1999). The influence of nectar and pollen availability on pollen transfer by individual flowers of oil-seed rape (*Brassica napus*) when pollinated by bumblebees (*Bombus lapidarius*. J. Ecol.

[b16] Cresswell JE (2011). A meta-analysis of experiments testing the effects of a neonicotinoid insecticide (imidacloprid) on honey bees. Ecotoxicology.

[b17] Dainat B, Evans JD, Chen YP, Gauthier L, Neumann P (2012). Dead or alive: deformed wing virus and *Varroa destructor* reduce the life span of winter honeybees. Appl. Environ. Microbiol.

[b18] De Valpine P, Hastings A (2002). Fitting population models incorporating process noise and observation error. Ecol. Monogr.

[b19] Desneux N, Decourtye A, Delpuech JM (2007). The sublethal effects of pesticides on beneficial arthropods. Annu. Rev. Entomol.

[b20] Gill RJ, Ramos-Rodriguez O, Raine NE (2012). Combined pesticide exposure severely affects individual- and colony-level traits in bees. Nature.

[b21] Goulson D, Lye GC, Darvill B (2008). Decline and conservation of bumble bees. Annu. Rev. Entomol.

[b22] Henry M, Béguin M, Requier F, Rollin O, Odoux J-F, Aupinel P (2012). A common pesticide decreases foraging success and survival in honey bees. Science.

[b23] Higes M, Martín-Hernández R, Botías C, Bailón EG, González-Porto AV, Barrios L (2008). How natural infection by *Nosema ceranae* causes honeybee colony collapse. Environ. Microbiol.

[b24] Holzschuh A, Dormann CF, Tscharntke T, Steffan-Dewenter I (2011). Expansion of mass-flowering crops leads to transient pollinator dilution and reduced wild plant pollination. Proc. R. Soc. B.

[b25] Iqbal J, Mueller U (2007). Virus infection causes specific learning deficits in honeybee foragers. Proc. R. Soc. B.

[b26] Ives AR, Einarsson A, Jansen VAA, Gardarsson A (2008). High-amplitude fluctuations and alternative dynamical states of midges in Lake Myvatn. Nature.

[b27] Khoury DS, Myerscough MR, Barron AB (2011). A quantitative model of honey bee colony population dynamics. PLoS ONE.

[b28] Khoury DS, Barron AB, Myerscough MR (2013). Modelling food and population dynamics in honey bee colonies. PLoS ONE.

[b29] Klein AM, Vaissiere BE, Cane JH, Steffan-Dewenter I, Cunningham SA, Kremen C (2007). Importance of pollinators in changing landscapes for world crops. Proc. R. Soc. B.

[b30] Kremen C, Williams NM, Aizen MA, Gemmill-Herren B, LeBuhn G, Minckley R (2007). Pollination and other ecosystem services produced by mobile organisms: a conceptual framework for the effects of land-use change. Ecol. Lett.

[b31] Krischik VA, Landmark AL, Heimpel GE (2007). Soil-applied imidacloprid is translocated to nectar and kills nectar-feeding *Anagyrus pseudococci* (Girault) (Hymenoptera: Encyrtidae). Environ. Entomol.

[b32] May RM (1977). Thresholds and breakpoints in ecosystems with a multiplicity of stable states. Nature.

[b33] Mayack C, Naug D (2009). Energetic stress in the honeybee *Apis mellifera* from *Nosema ceranae* infection. J. Invertebr. Pathol.

[b34] Moret Y, Schmid-Hempel P (2000). Survival for immunity: the price of immune system activation for bumblebee workers. Science.

[b35] Müller CB, Schmid-Hempel P (1992). Variation in life-history pattern in relation to worker mortality in the bumblebee, *Bombus lucorum*. Funct. Ecol.

[b36] Mumby PJ, Steneck RS, Hastings A (2013). Evidence for and against the existence of alternate attractors on coral reefs. Oikos.

[b37] Oldroyd BP (2007). What’s killing American honey bees?. PLoS Biol.

[b38] Potts SG, Biesmeijer JC, Kremen C, Neumann P, Schweiger O, Kunin WE (2010). Global pollinator declines: trends, impacts and drivers. Trends Ecol. Evol.

[b39] Ratnieks FLW, Carreck NL (2010). Clarity on honey bee collapse?. Science.

[b40] Rueppell O, Kaftanouglu O, Page RE (2009). Honey bee (*Apis mellifera*) workers live longer in small than in large colonies. Exp. Gerontol.

[b41] Schafer MO, Ritter W, Pettis JS, Neumann P (2011). Concurrent parasitism alters thermoregulation in honey bee (Hymenoptera: Apidae) winter clusters. Ann. Entomol. Soc. Am.

[b42] Schmid-Hempel P (1998). Parasites in Social Insects.

[b43] Schmid-Hempel P, Heeb D (1991). Worker mortality and colony development in bumblebees, *Bombus lucorum* (L.) (Hymenoptera, Apidae). Mitt. Schweiz. Entomol. Ges.

[b44] Schneider CW, Tautz J, Grünewald B, Fuchs S (2012). RFID tracking of sublethal effects of two neonicotinoid insecticides on the foraging behavior of *Apis mellifera*. PLoS ONE.

[b45] Sumpter DJT, Martin SJ (2004). The dynamics of virus epidemics in Varroa-infested honey bee colonies. J. Anim. Ecol.

[b46] Thompson HM (2001). Assessing the exposure and toxicity of pesticides to bumblebees (*Bombus* sp.). Apidologie.

[b47] Vanbergen AJ, The Insect Pollinators Initiative (2013). Threats to an ecosystem service: pressures on pollinators. Front. Ecol. Environ.

[b48] Whitehorn PR, O’Connor S, Wackers FL, Goulson D (2012). Neonicotinoid pesticide reduces bumble bee colony growth and queen production. Science.

[b49] Williamson SM, Wright GA (2013). Exposure to multiple cholinergic pesticides impairs olfactory learning and memory in honeybees. J. Exp. Biol.

[b50] Winfree R, Williams NM, Gaines H, Ascher JS, Kremen C (2008). Wild bee pollinators provide the majority of crop visitation across land-use gradients in New Jersey and Pennsylvania, USA. J. Appl. Ecol.

