# Supplementary material for: Chronic sublethal stress causes bee colony failure
Source: Ecol Lett. 2013 Oct 6;16(12):1463–9. doi: 10.1111/ele.12188 (PMC4299506; doi:10.1111/ele.12188)
Supplement: Supplementary file 1 [file ele0016-1463-sd1.pdf]

Chronic sublethal stress causes bee colony failure

Supplementary Information

John Bryden[1*], Richard J. Gill[1,2], Robert A. A. Mitton[1], Nigel E. Raine[1], Vincent A. A. Jansen[1]

[1] School of Biological Sciences, Royal Holloway University of London, Egham, Surrey, TW20 0EX, UK.

[2] Current address: Department of Life Sciences, Imperial College London, Silwood Park, Ascot, Berkshire, SL5 7PY, UK.

* email: john.bryden@rhul.ac.uk

Section 1. Materials and Methods

1.1.) Colony setup

Sixteen *Bombus terrestris* colonies (supplied by Syngenta Bioline, Weert, the Netherlands) were used in the experiment. On the day of arrival we ranked the 16 colonies according to size determined by the total number of workers and pupae. The largest two colonies were randomly assigned to control and neonicotinoid (imidacloprid) pesticide treatment. This process was repeated for the second largest pair of colonies, and so on for each consecutive pair (*n* colonies = 8 control and 8 neonicotinoid).

Each colony was housed in a bipartite wooden nest box (28 × 16 × 11 cm): the rear chamber containing the brood (henceforth the 'brood chamber') and a front chamber housing a gravity feeder containing sucrose solution and a pollen dish. A hole in the partition allowed bees to move freely between the two chambers. Each chamber had a hinged transparent Perspex lid for observation. Colonies were kept at room temperature in a naturally lit laboratory throughout the experiment (although the brood chamber of each colony was covered with cardboard when not being observed to mimic the darkness of a subterranean nest).

1.2.) Pesticide treatment and feeding regime

All colonies were provided with fresh 40/60% sucrose/water (v/v) solution every two days (or three days over the weekend), with 10ml provided in the first week and an incremental increase of 2ml for each subsequent week (therefore by the sixth, and last, experimental week colonies were provided with 20ml). Before feeders were refilled we disposed of the remaining sucrose solution and rinsed the feeder with water.

For the pesticide treatment, we dissolved imidacloprid ($C_9H_{10}ClN_5O_2$ powder; grade: PESTANAL®, analytical standard; brand: Fluka) in acetone to produce a primary stock solution (1mg/ml). An aliquot of the primary stock solution was then added to a 40/60% sucrose/water (v/v) solution to produce a 10μg/L (10 ppb) imidacloprid solution. A control solution was made by repeating this process but using an acetone stock solution (i.e. without imidacloprid).

All colonies were provided with a specified weight of defrosted honeybee-collected pollen (Koppert B.V., Berkel en Rodenrijs, Netherlands) in a plastic dish (depth = 2.2cm, diameter = 4cm) three times per week (provided at the same time as sucrose feeders were refilled). Before refilling the pollen dish we disposed of the old pollen and thoroughly rinsed the dish with water. Each colony was provided with 1g of pollen per refill during weeks 1–3 (i.e., 3g per week), which was increased to 2g of pollen per refill during weeks 4–6 (i.e., 6g per week) to account for the greater demand of larger colonies.

1.3.) Experimental measurements

All workers present in the colony before the experimental treatment started (their precise age unknown) were individually tagged with a uniquely numbered Opalith tag (Opalith Plättchen, Christian Graze KG, Weinstadt-Endersbach, Germany). Throughout the 42-day experiment all colonies were inspected daily for five days of the week (Monday-Friday) to record the number of newly eclosed workers (any workers found on Monday morning were assumed to have eclosed with equal probability on Saturday or Sunday) and the number of adult workers and larvae that had died. All workers eclosing during the experiment were each tagged with a uniquely numbered and coloured Opalith tag within 2 days of eclosion (thus age was known). Any dead individuals (larvae, workers and queen) found in a colony were removed and frozen (at -20°C). Similarly any gynes (unmated newly eclosed queens) and males that eclosed during the experiment were removed and frozen (at -20°C) when found. At the end of the 42nd day of experimental treatment all colonies were frozen (at -20°C).

Section 2. Model Analysis

We analysed Equations (1) and (2) by substituting

$$N = S + cI$$

$$P = S/N$$

into the equations, where N denotes the effective size of the colony and P is the proportion of susceptible individuals. This gives,

$$\frac{dN}{dt} = N\left(b - \frac{\mu}{N+\phi} - \beta(1-c)P - \nu(1-P)\right) \qquad (3)$$

$$\frac{dP}{dt} = b - P[b + \beta - \beta(1-c)P - \nu(1-P)]\,. \qquad (4)$$

Solving these two equations for equilibrium we find that,

$$\tilde{N} = \frac{\mu}{b-\beta(1-c)\tilde{P}-\nu(1-\tilde{P})} - \phi \qquad (5)$$

$$\tilde{P} = \frac{b+\beta-\nu-\sqrt{b^2+(\beta-\nu)^2+2b(\nu+\beta(2c-1))}}{2\beta(1-c)-2\nu}\,. \qquad (6)$$

Looking at Equation (3), we can see that N is increasing when $N > \widetilde{N}$, and it is decreasing when $N < \widetilde{N}$. However, Equation (6) shows that the proportion of susceptible bees P will always tend to equilibrium, independent of N. This means there is a saddle point in the dynamics of Equations (1) and (2) which generates multiple outcomes of success and failure.

2.1.) Changes in the saddle point coordinates lead to three different types of dynamics in the model

The coordinates of the saddle-point depend on the relationship of the birth rate b to the values of the other parameters. Manipulation of Equations (5) and (6) shows there are two thresholds for b,

$$T_{grow} = \frac{(\mu + \beta\phi)(\mu + \nu\phi)}{\phi(\mu + (\beta c + \nu)\phi)}$$

$$T_{fail} = \beta\nu/(\beta c + \nu)\ .$$

When $b > T_{grow}$ all colonies will grow, when $b < T_{fail}$ all colonies will fail (at $b = T_{fail}$, $\widetilde{N} \to \infty$), and finally when $T_{fail} < b < T_{grow}$ there is a saddle point in the dynamics. Changing the other parameters moves the thresholds (see Figure S1).

2.2.) Multiple outcomes in the dynamics crucially depend on the non-linear per capita death rate

The saddle-point creates a switch in the dynamics of N, see Equation (3). Simple manipulation shows how this switch in the dynamics requires the positive density dependence term in Equations (1) and (2): $\frac{\mu}{N+\phi}S$. When we remove this term or replace it with a linear term μ, there is no longer a point $\widetilde{N}$ where $\frac{dN}{dt} = 0$, meaning there is no saddle point and hence no multiple outcomes in the dynamics. When we replace it with a linear term μ (see SLS Variant Model in the main text), simple linear stability analysis shows there is a threshold on the value of b between colony success and failure at:

$$T_{fail} = \frac{(\beta + \mu)(\mu + \nu)}{\beta c + \mu + \nu}$$

Supplementary Figures

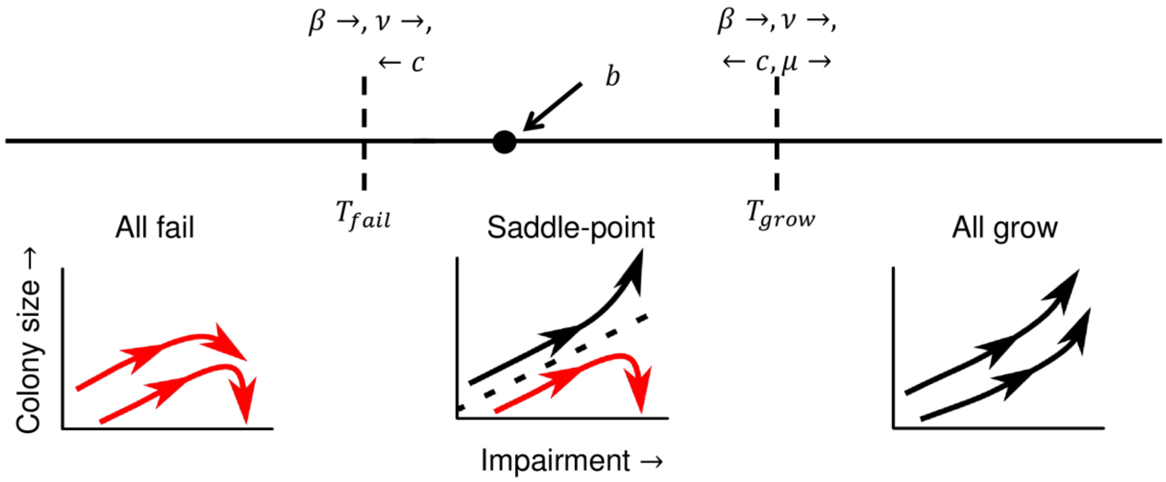

Figure S1. Schematic diagram summarising the relationship of the parameter b (birthrate) to the other parameters. Increasing β (impairment rate) or ν (mortality rate of impaired bees), or decreasing c (contribution of impaired bees) will move thresholds T_{fail} and T_{grow} to the right. Increasing μ (mortality rate due to poor colony function) will increase the range over which the saddle-points are present.

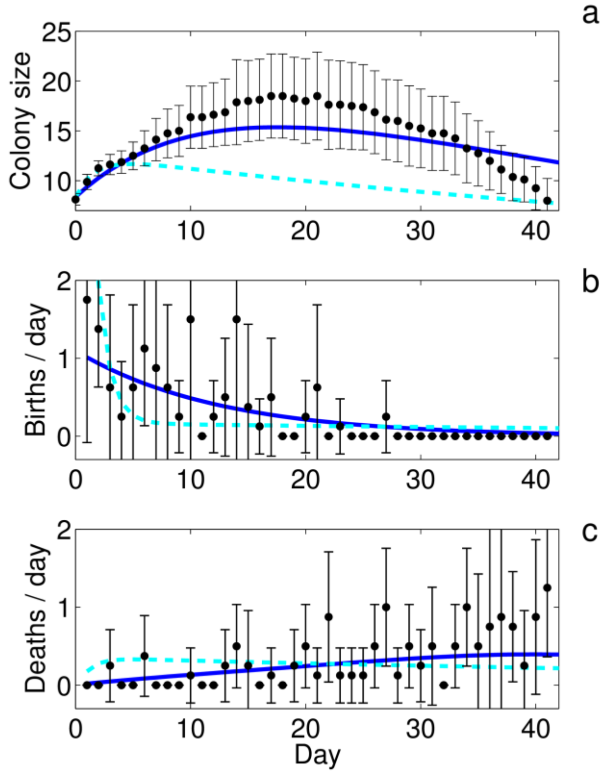

Figure S2. Comparison of the best fits, against the data from the treatment colonies, of the SLS Model (blue line) and LA Model (dashed cyan line). **a**, The SLS Model fits the dynamic better than the LA Model. **b, c** The fitting was done against birth and death rates. Parameters for Lagged Model: $g = 0.0103, \gamma = 0.600,\ \lambda = 8.11 \times 10^{-13}, \xi = 0.0221, initial\_larvae\_proportion\ =\ 0.519$.

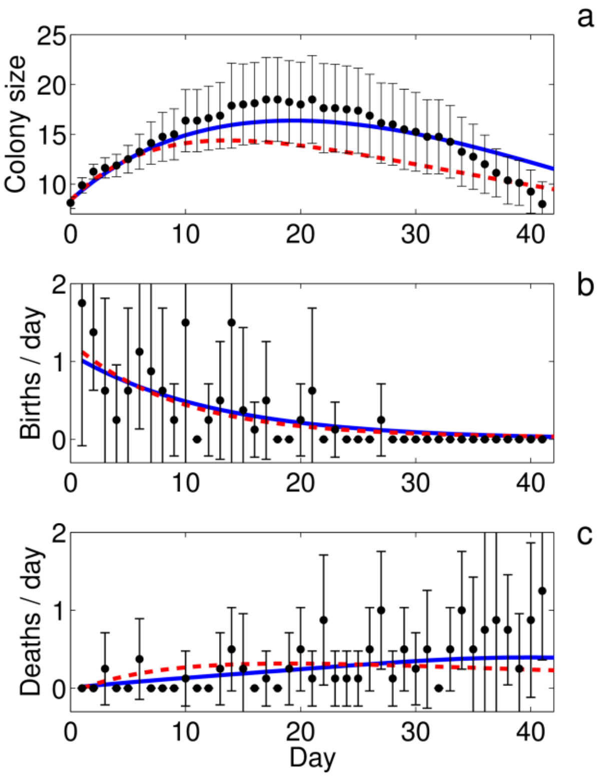

Figure S3. Comparison of the best fits, against the data from the treatment colonies, of the SLS Model (blue line) and SLS Variant Model (dashed red line). **a**, The SLS Model fits the dynamic better than the SLS Variant Model model. **b, c** The fitting was done against birth and death rates. Parameters for SLS Variant Model: $\beta = 0.242, b = 0.141, c = 0.00699, \mu = 3.04 \times 10^{-11}, \nu = 0.0255$.

Supplementary Tables

Table 1. Initial sizes of the control colonies

Colony	1	2	3	4	5	6	7	8
Number of workers	2	8	7	6	10	6	6	7

Table 2. Cumulative worker production of the control colonies

Colony<br>Day	1	2	3	4	5	6	7	8
1	0	0	0	0	0	3	0	0
2	0	0	1	0	4	6	3	1
3	0	0	2	0	4	8	3	2
4	0	0	2	0	9	9	4	2
5	0	0	2	0	11	9	6	3
6	0	0	2	1	14	9	8	4
7	0	1	2	2	17	10	11	6
8	1	1	2	3	24	16	13	7
9	1	1	2	3	26	18	14	8
10	1	1	5	3	30	20	15	8
11	1	1	5	5	30	21	15	8
12	1	1	5	5	30	21	15	8
13	1	1	5	5	30	22	15	9
14	1	1	5	6	30	24	16	10
15	1	1	5	6	30	27	17	11
16	1	1	5	8	31	28	17	11
17	1	2	5	8	31	35	18	14
18	1	3	5	8	31	38	19	19
19	1	4	5	8	31	38	19	20
20	1	6	5	9	31	39	20	21
21	1	9	5	10	31	41	21	23
22	1	10	5	10	31	42	22	27
23	1	10	6	12	31	43	22	28
24	1	14	9	18	31	43	24	30
25	1	14	10	18	31	46	26	32
26	1	14	11	20	32	49	28	34
27	1	15	12	23	34	52	30	36
28	1	17	14	27	37	56	33	39
29	1	18	14	29	39	57	34	40
30	1	21	15	30	39	61	34	44
31	1	21	16	31	39	66	34	45
32	1	27	17	31	39	66	34	46
33	1	29	19	35	39	66	34	46
34	1	32	21	40	39	67	35	47
35	1	36	24	46	39	68	37	48
36	1	40	28	48	41	72	39	48
37	1	45	30	48	43	76	42	50

38	1	49	30	48	44	79	44	50
39	1	51	33	48	52	80	47	51
40	1	52	35	49	54	80	48	51
41	1	54	37	51	57	80	50	52
42	1	57	40	54	61	80	52	54

Table 3. Cumulative mortality of the control colonies

Colony Day	1	2	3	4	5	6	7	8
1	0	0	0	2	1	0	2	1
2	0	0	0	2	1	0	2	1
3	0	0	1	2	1	1	2	1
4	0	0	1	2	1	1	2	1
5	0	0	1	2	1	1	2	1
6	0	0	1	2	1	2	2	1
7	0	0	1	2	2	4	2	1
8	0	0	1	2	2	5	3	1
9	0	0	1	3	4	5	3	1
10	0	0	1	3	4	5	3	1
11	0	0	1	3	4	5	3	1
12	0	0	1	3	4	5	3	1
13	0	0	1	3	4	5	3	2
14	0	1	2	4	4	5	3	4
15	0	1	2	4	4	5	3	4
16	0	1	2	4	4	5	3	4
17	1	1	2	4	5	5	3	4
18	1	1	2	4	5	6	3	4
19	1	1	2	4	5	6	3	4
20	1	1	2	4	5	6	3	4
21	1	1	2	4	5	6	4	5
22	1	3	2	4	5	6	5	5
23	1	3	2	4	5	6	6	6
24	1	3	2	4	5	6	6	6
25	1	3	2	4	5	6	6	6
26	1	3	2	4	9	9	6	6
27	1	3	2	4	14	13	7	7
28	1	3	3	4	20	17	8	9
29	1	3	3	4	20	17	8	9
30	1	4	3	4	20	17	9	9
31	1	4	3	4	20	17	10	9
32	1	4	4	4	22	18	10	9
33	1	4	4	4	22	18	10	9
34	1	4	4	4	23	18	10	10
35	1	4	5	4	25	18	11	12
36	1	4	5	6	25	18	12	14
37	1	5	5	7	26	18	12	15

38	1	6	5	8	27	19	13	16
39	1	6	6	10	27	20	16	16
40	1	6	6	10	27	20	16	18
41	1	6	7	10	27	21	17	20
42	2	6	8	11	27	22	18	23

Table 4. Initial sizes of the treatment colonies

Colony	1	2	3	4	5	6	7	8
Number of workers	4	13	8	2	9	16	5	9

Table 5. Cumulative worker production of the treatment colonies

Colony<br>Day	1	2	3	4	5	6	7	8
1	0	0	1	0	0	1	3	1
2	0	0	1	3	5	2	5	4
3	2	1	3	4	7	4	6	4
4	2	1	3	7	9	4	6	4
5	2	1	3	7	11	4	6	4
6	2	1	4	8	14	4	6	4
7	2	1	5	10	17	5	7	5
8	2	1	5	10	21	8	7	5
9	2	1	5	10	24	9	8	5
10	2	1	5	10	25	10	8	5
11	4	1	5	10	30	12	11	5
12	4	1	5	10	30	12	11	5
13	5	1	5	10	31	12	11	5
14	7	1	5	11	32	12	11	5
15	7	1	7	11	41	12	11	6
16	7	1	7	11	41	12	14	6
17	7	1	7	11	41	12	15	6
18	7	1	8	11	42	12	17	6
19	7	1	8	11	42	12	17	6
20	7	1	8	11	42	12	17	6
21	7	1	8	12	43	12	17	6
22	7	1	8	12	44	13	20	6
23	7	1	8	12	44	13	20	6
24	7	1	8	12	44	13	20	7
25	7	1	8	12	44	13	20	7
26	7	1	8	12	44	13	20	7
27	7	1	8	12	44	13	20	7
28	7	1	9	12	44	13	21	7
29	7	1	9	12	44	13	21	7
30	7	1	9	12	44	13	21	7
31	7	1	9	12	44	13	21	7
32	7	1	9	12	44	13	21	7
33	7	1	9	12	44	13	21	7
34	7	1	9	12	44	13	21	7
35	7	1	9	12	44	13	21	7
36	7	1	9	12	44	13	21	7
37	7	1	9	12	44	13	21	7
38	7	1	9	12	44	13	21	7
39	7	1	9	12	44	13	21	7

40	7	1	9	12	44	13	21	7
41	7	1	9	12	44	13	21	7
42	7	1	9	12	44	13	21	7

Table 6. Cumulative mortality of the treatment colonies

Colony<br>Day	1	2	3	4	5	6	7	8
1	0	2	0	0	2	0	0	1
2	0	2	0	0	2	0	0	1
3	0	2	0	0	2	0	0	1
4	0	3	0	0	3	0	0	1
5	0	3	0	0	3	0	0	1
6	0	3	0	0	3	0	0	1
7	1	3	0	0	3	1	1	1
8	1	3	0	0	3	1	1	1
9	1	3	0	0	3	1	1	1
10	1	3	0	0	3	1	1	1
11	1	4	0	0	3	1	1	1
12	1	4	0	0	3	1	1	1
13	1	4	0	0	3	1	1	1
14	2	4	0	0	3	1	1	2
15	3	5	0	0	4	1	1	3
16	3	5	0	0	6	1	1	3
17	3	5	0	0	6	1	1	3
18	3	5	0	1	6	1	1	3
19	3	5	0	1	6	1	1	3
20	3	5	0	1	7	1	1	4
21	3	6	0	2	8	1	1	5
22	3	6	0	2	8	1	2	5
23	3	8	1	2	9	3	2	6
24	3	8	1	2	9	3	3	6
25	3	8	1	2	9	3	4	6
26	3	8	1	2	10	3	4	6
27	3	8	2	2	11	4	5	6
28	3	9	4	3	13	5	6	6
29	3	10	4	3	13	5	6	6
30	4	10	5	3	14	6	6	6
31	4	10	6	3	15	6	6	6
32	4	10	6	3	17	7	7	6
33	4	10	6	3	17	7	7	6
34	4	10	6	4	18	8	7	7
35	5	10	6	6	20	9	8	8
36	5	10	6	8	22	9	8	8
37	5	10	6	8	26	11	8	8
38	5	10	6	8	32	11	9	8
39	6	10	7	9	33	13	9	8

40	6	10	7	9	35	13	9	8
41	7	10	8	9	38	14	9	9
42	8	11	9	10	41	15	9	11
